# Morphological analysis for subaxial cervical pedicle screw insertion in developmental and non-developmental canal stenosis

**DOI:** 10.1186/s12891-019-2577-1

**Published:** 2019-05-10

**Authors:** Song Wang, Gangyi Yang, Ce Zhu, Jianping Kang, Qing Wang

**Affiliations:** 1 Department of Spine Surgery, the Affiliated Hospital of Southwest Medical University, No. 25 Taiping St., Luzhou, 646000 Sichuan China; 2 Department of Orthopedics, Mianyang Central Hospital, Mianyang, 621000 Sichuan China

**Keywords:** Developmental canal stenosis, Subaxial cervical spine, Pedicle screw, Pedicle morphology, Parameter, Computed tomography

## Abstract

**Background:**

This study aimed to evaluate the safety and feasibility of subaxial cervical pedicle screw (CPS) insertion by comparing the morphological parameters between developmental canal stenosis (DCS) and non-developmental canal stenosis (NDCS) patients.

**Methods:**

A total of 120 Chinese patients who had undergone cervical spinal multiplanar CT imaging from September 2010 to December 2014 were included in this study. According to the Pavlov ratio (PR), participants were classified into a DCS group (PR < 0.82) and an NDCS group (PR ≥0.82). CT reconstruction images of the cervical pedicles from C3 to C7 were selected for further analysis, and detailed morphological parameters for subaxial CPS insertion including pedicle outer width (POW), tiny cervical pedicle (TCP), pedicle transverse angle (PTA), and range of safe angle (RSA) were measured and compared in these two groups.

**Results:**

A total of 600 images (1200 pedicles) from these 120 patients were measured. The POW in the DCS group was wider than that in the NDCS group at each level, while the number of TCPs in the DCS group was significantly less than that in the NDCS group at the C3, C4, and C5 vertebrae. There was no significant difference in PTA at any level between the two groups, however the RSA in the DCS group was greater than that in the NDCS group from C4 to C7.

**Conclusions:**

Subaxial CPS for DCS patients may be safer and more feasible than that for NDCS patients. However, as the subaxial cervical pedicle is relatively small, CPS insertion is difficult and preoperative CT evaluation is recommended for both DCS and NDCS patients.

## Background

Developmental canal stenosis (DCS) has been described as one of the primary risk factors responsible for spinal cord compression syndrome, with some reports of high rates of DCS among patients with cervical myelopathy [1–3]. Those patients with severe myelopathy may require cervical operation via an anterior/posterior or a combined approach. Currently, posterior cervical spinal instrumentation has enabled spinal cord decompression and fixation of the cervical spine using a single approach [4, 5]. However, it is essential that any injury to the spinal cord or the vertebral artery be avoided when the pedicle screw is inserted [6–10].

Successful placement of pedicle screws in the subaxial cervical spine requires a sufficient three-dimensional understanding of pedicle morphology. Several cadaveric and a few radiological anatomical studies of the cervical pedicle have been reported [11–15]. However, there are few reports of comparisons of subaxial cervical pedicle morphology between DCS and NDCS patients. In 2010, Miyazaki et al. [16] compared the morphology of DCS and NDCS patients and reported that posterior screw insertion should be more carefully in DCS patients than in NDCS patients. In contrast with this report, however, our surgical team has performed subaxial cervical pedicle screw (CPS) insertion for indicated patients since 2006 [17] and has found that CPS insertion in DCS patients was not more difficult in practice than in NDCS patients. This suggested that additional in-depth measurements of these two patient groups were warranted.

In this study, we measured and calculated the morphological parameters in DCS and NDCS patients based on multiplanar reconstruction images of computed tomography (CT), and tried to evaluate the safety and feasibility of subaxial CPS insertion in these two patient groups.

## Methods

### Participants

From September 2010 to December 2014, 120 consecutive Chinese patients that underwent cervical spinal multiplanar CT imaging were enrolled in this study. The enrolled patients had cervical spondylotic myelopathy (CSM), cervical spondylotic radiculopathy (CSR), or cervical spinal cord injury without fracture and dislocation (CSIWFD). Subjects with evidence of congenital abnormalities, infection, tumors, or cervical fractures and dislocations were excluded. The present study was conducted in accordance with the declaration of Helsinki and with approval from the Ethics Committee of the Affiliated Hospital of Southwest Medical University. All patients provided written informed consent prior to their inclusion in this study.

### Classification of study groups

**Fig. 1:**
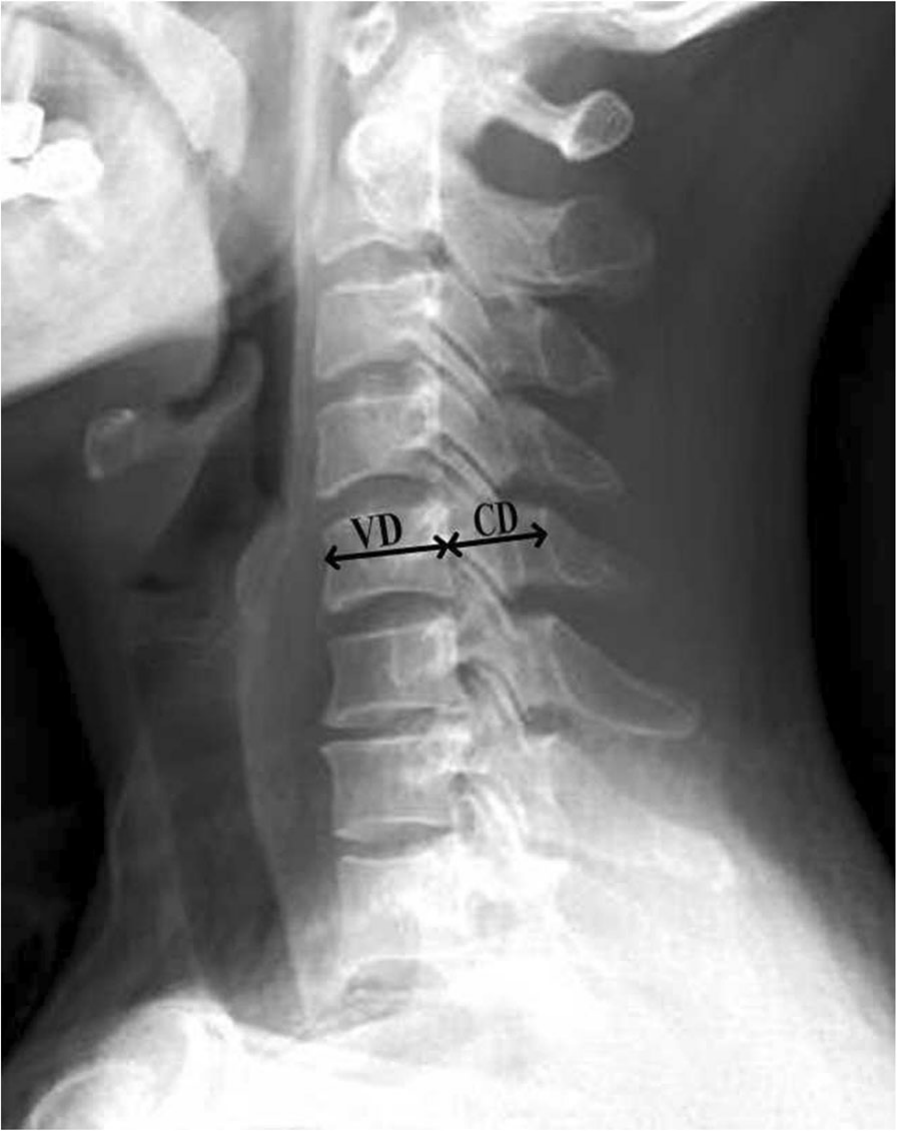
Measurement of the Pavlov ratio on lateral radiography. VD indicated the vertebral diameters, namely, the cervical sagittal diameter of the vertebral body at the midpoint; CD, the canal diameter, namely, the sagittal diameter of the spinal canal from the posterior surface of the vertebral body to the nearest point of the corresponding spinal laminar line. The Pavlov ratio was determined with the formula CD/VD in every level [18]

The participants were classified into two groups according to the Pavlov ratio, based on ratio of the sagittal diameter of the spinal canal to the sagittal diameter of the middle vertebral body [18]. To establish this ratio, cervical spinal lateral radiographs were imported into a picture archiving and communication system (PACS) (MedPACS, HUAHAI Medical Information Technology Co. Ltd., Xi’an, China). The cervical sagittal mid-vertebral diameters (VD) and the sagittal spinal canal diameter (CD) from C3 to C7 were then measured (Fig. 1). The Pavlov ratio was determined in every level according to the formula Pavlov ratio = CD/VD. Patients with a Pavlov ratio < 0.82 were enrolled in the DCS group, while those with a ratio of 0.82 or greater were enrolled in the NDCS group.

### CT scanning protocol and measurements

Cervical CT scans were performed using a multi-slice scanner (Light speed VCT, GE Healthcare, Indiana, IN). Patients were placed in the supine position and with the neck in the neutral position. Primary images were obtained in 0.625 mm slices from the C1 to C7 levels. Image multiplanar reconstructions were then generated using a CT workstation (Advantage Workstation, version AW4.0, GE Healthcare, Indiana, IN) and transverse images parallel to the endplates of the vertebral body were reconstructed. Then, images of the cervical pedicles at the widest slice from C3 to C7 were selected, and 600 images were measured in the PACS.

**Fig. 2:**
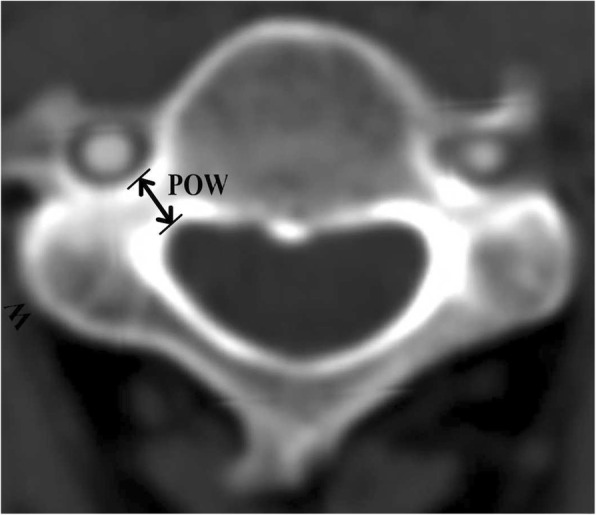
Measurement of pedicle outer width (POW) on transverse reconstruction images of computed tomography. POW indicated the mediolateral diameter of the pedicle isthmus, perpendicular to the pedicle axis

**Fig. 3:**
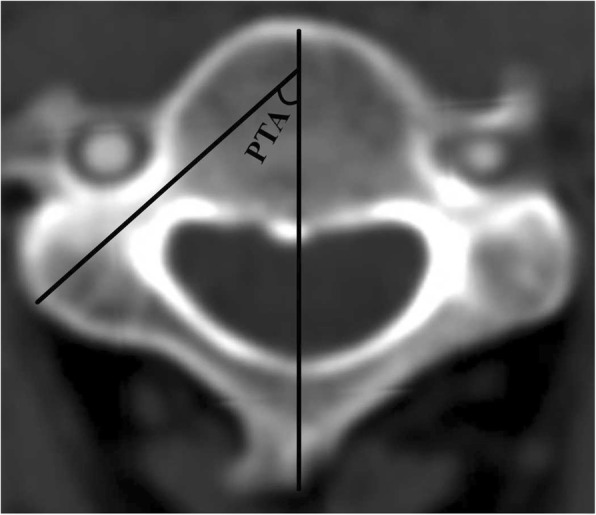
Measurement of pedicle transverse angle (PTA). PTA indicated the angle between the pedicle axis and the midline of the vertebral body

**Fig. 4:**
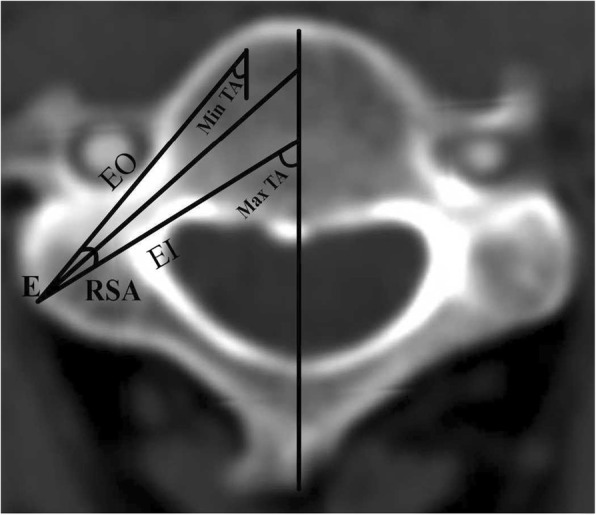
Measurement of range of safe angle (RSA). RSA indicated the difference of the Max TA and the Min TA, namely, the intersection angle between EI and EO. EI was the line crossing the entry point (E) and the inner cortex in the pedicle isthmus. EO was the line crossing the point E and the outer cortex in the pedicle isthmus. The point E was the crossing point of the pedicle axis and the surface cortex of the pedicle. The Max TA was the angle between the line EI and the midline of the vertebral body. The Min TA was the angle between the line EO and the midline of the vertebral body (The diameter of pedicle screw was neglected)

The definitions and measurements of morphological parameters were as follows:

1. Pedicle outer width (POW) was the mediolateral diameter of the pedicle isthmus, perpendicular to the pedicle axis (Fig. 2).
2. Tiny cervical pedicle (TCP) was apedicle of POW < 4 mm. A pedicle of 4 mm in diameter was assumed to be the smallest one compatible with the insertion of a 3.5 mm diameter cervical pedicle screw [15], and anything smaller was therefore termed TCP. TCPs at every level in each group were counted.
3. Pedicle transverse angle (PTA) was the angle between the pedicle axis and the midline of the vertebral body (Fig. 3).
4. Range of safe angle (RSA) was the difference between the maximum transverse angle (Max TA) and the minimum transverse angle (Min TA). The Max TA was the angle between the line (EI) crossing the entry point (E) and the inner cortex in the pedicle isthmus and the midline of the vertebral body. The Min TA was the angle between the line (EO) crossing point E and the outer cortex in the pedicle isthmus and the midline of the vertebral body (the diameter of pedicle screw was not considered). The entry point E was considered as being the intersection point of the pedicle axis and the surface cortex of the pedicle. The RSA was the angle formed by the intersection of the EI and EO lines (Fig. 4).

### Measurement agreement

All measurements were performed independently by two clinicians, who were attending in the spine surgery department. The CT images were selected and numbered using random numbers generated using Excel 2003 (Microsoft Corp., Redmond, WA, USA) by the study designer. To ensure that the axial slice selected was indeed truly at the widest portion of the pedicle, the two trained clinicians made their decisions simultaneously. In cases where their opinions differed, adjacent slices were measured in the workstation to determine the widest slice. These numbered imaged were then measured by the two observers independently according to number order (left and right pedicles were measured simultaneously). Two weeks later, all the images were numbered and measured again.

### Statistical analysis

All statistical analyses were performed using the Statistical Package for Social Sciences (SPSS) software (version 22.0; SPSS Inc., Chicago, IL, USA). Values are represented as means ± standard deviations. Student’s t-tests (two tailed) were used to analyze numerical data, while Pearson’s chi-squared tests were used to analyze categorical data. The intra- and inter-observer correlations were analyzed by intraclass correlation coefficient (ICC) to ensure good reproducibility between the measures obtained by these two observers. *P* < 0.05 was the threshold of statistical significance.

## Results

A total of 120 patients (74 males and 46 females) were enrolled in this study, with an average age of 52.2 years (range: 26–74 years). The baseline characteristics of the two study groups (DCS and NDCS groups) were compared, and no significant differences in the number, sex, age, or diseases of the subjects were detected between groups (*P* > 0.05). As expected, however, the Pavlov ratio in the DCS group was less than in the NDCS group (*P* < 0.05) (Table 1).

**Table 1:** Baseline data between the study groups based on the Pavlov ratio

Groups	Number Subjects	Sex (Male/Female)	Age	The Pavlov ratio	Disease
DCS	60	36/24	53.5 ± 13.9	0.68 ± 0.09	CSM 38 CSIWFD 22
NDCS	60	38/22	50.8 ± 11.6	0.96 ± 0.11*	CSR 40 CSM 15 CSIWFD 5

*DCS* Developmental canal stenosis, *NDCS* Non-developmental canal stenosis, *CSM* Cervical spondylotic myelopathy, *CSIWFD* Cervical spinal cord injury without fracture and dislocation, *CSR* Cervical spondylotic radiculopathy

* Independent Samples T Test, *P* < 0.05

A total of 600 images (1200 pedicles) were measured from C3 to C7. Averages and standard deviations of the linear and angular parameters were calculated at each level. The intra- and inter-observer ICC values were 0.92 and 0.85, respectively. An intra- and inter-observer variability analysis was performed to ensure good correlation between the measures obtained by the two observers. The averages of these measurements were used for the final data analysis. Comparisons of morphological parameters between patients in the DCS and NDCS groups are shown in Table 2.

**Table 2:** Comparisons of morphological parameters between the two groups

Level	Group	POW(mm)			TCP(number)			PTA(°)			RSA(°)		
		Male	Female	Total	Male	Female	Total	Male	Female	Total	Male	Female	Total
C3	DCS	5.7 ± 0.6	4.8 ± 0.8	5.3 ± 0.7	2	5	7 (11.6%)	43.7 ± 3.4	44.4 ± 3.6	43.9 ± 3.5	10.1 ± 3.7	8.9 ± 2.8	9.3 ± 3.3
	NDCS	4.7 ± 0.8	3.7 ± 1.6	4.1 ± 0.9*	8	13	21 (35.0%)#	44.5 ± 3.2	46.1 ± 3.5	45.5 ± 3.4	9.3 ± 3.6	8.2 ± 2.8	8.7 ± 3.2
C4	DCS	5.8 ± 0.6	5.0 ± 0.7	5.5 ± 0.8	3	5	8(13.3%)	47.2 ± 3.4	48.2 ± 3.4	47.6 ± 3.4	11.9 ± 3.5	10.7 ± 4.6	11.3 ± 3.8
	NDCS	4.9 ± 1.1	4.0 ± 0.7	4.4 ± 0.8*	5	12	17(28.3%)#	47.2 ± 2.8	46.7 ± 3.6	46.9 ± 3.4	11.2 ± 2.6	9.8 ± 3.7	10.5 ± 3.2*
C5	DCS	6.0 ± 0.7	5.4 ± 0.6	5.8 ± 0.7	1	4	5(8.3%)	45.3 ± 4.3	45.6 ± 4.2	45.4 ± 4.3	13.1 ± 3.4	11.6 ± 2.7	12.3 ± 3.8
	NDCS	5.1 ± 1.0	4.4 ± 0.7	4.7 ± 0.9*	3	10	13(21.7%)#	47.1 ± 3.0	44.8 ± 4.0	45.7 ± 3.8	11.2 ± 3.6	10.3 ± 4.9	10.8 ± 4.2*
C6	DCS	6.5 ± 0.9	5.8 ± 0.6	6.2 ± 0.7	1	4	5(8.3%)	38.9 ± 4.4	41.7 ± 4.8	40.0 ± 4.7	13.5 ± 3.0	12.3 ± 3.8	12.9 ± 3.4
	NDCS	5.7 ± 1.1	4.7 ± 0.7	5.1 ± 0.9*	3	6	9(15.0%)	41.3 ± 4.5	42.6 ± 5.3	42.3 ± 4.2	11.8 ± 4.1	10.2 ± 4.6	11.0 ± 4.4*
C7	DCS	7.6 ± 0.4	6.5 ± 1.5	7.0 ± 0.8	None	None	None	33.4 ± 3.7	34.5 ± 4.8	33.8 ± 4.2	14.5 ± 3.2	12.6 ± 4.7	13.2 ± 3.9
	NDCS	6.7 ± 1.6	5.6 ± 0.7	6.1 ± 0.7*	None	None	None	35.1 ± 5.3	36.2 ± 3.7	35.9 ± 4.6	13.0 ± 3.1	11.7 ± 4.2	12.2 ± 3.9*

*POW* Pedicle outer width, *TCP* Tiny cervical pedicle (POW less than 4 mm), *PTA* Pedicle transverse angle, *RSA* Range of safe angle

* Independent Samples T Test, *P* < 0.05

# Pearson’s Chi Square Test, *P* < 0.05

### Pow

**Fig. 5:**
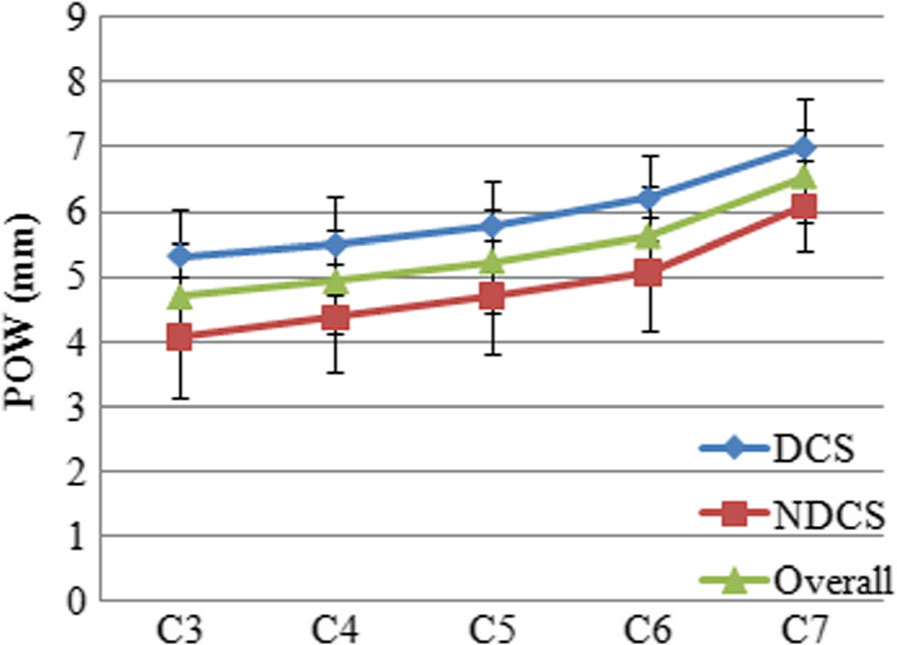
POW in the DCS and NDCS groups. POW indicated pedicle outer width; DCS, developmental canal stenosis; NDCS, non-developmental canal stenosis

The mean POW of C3 and C7 were 5.3 ± 0.7 mm and 7.0 ± 0.8 mm in the DCS group and 4.4 ± 0.8 mm and 6.1 ± 0.7 mm in the NDCS group (Table 2). As shown in Fig. 5, the mean POW tended to increase from C3 to C7 in the both groups. The smallest mean POW was found at C3 and the largest was at C7 in the both groups (Fig. 5). Comparison of the DCS and NDCS groups revealed the POW in the DCS group was significantly wider than that in the NDCS group at each level (*P* < 0.05). In each group, the POW of male was wider than the POW of female at each level (*P* < 0.05).

### TCP

**Fig. 6:**
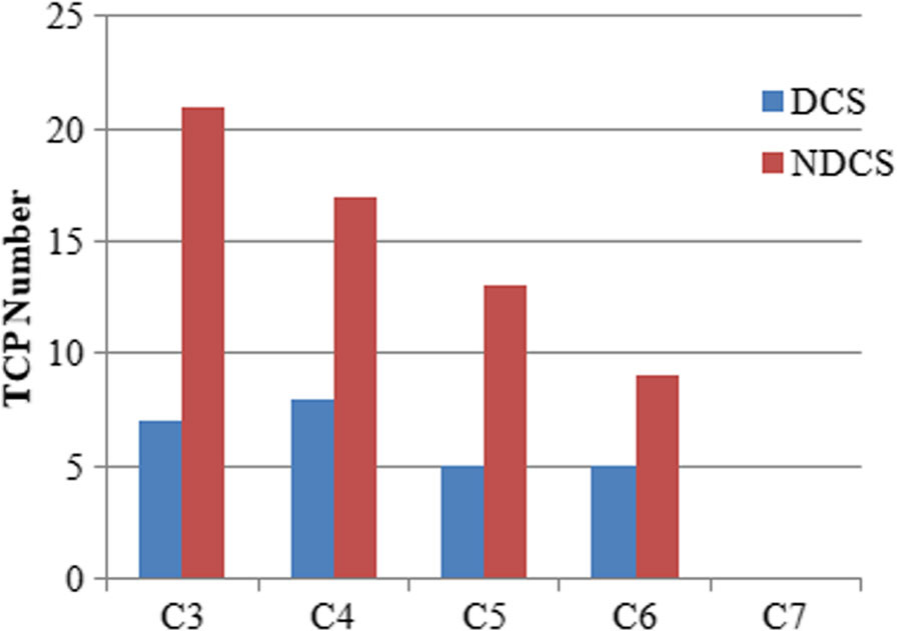
TCP in the DCS and NDCS groups. TCP indicated tiny cervical pedicle of POW less than 4 mm

The greatest number of TCPs was observed in C3, while none was observed in C7 in either group (Table 2). As shown in Fig. 6, the TCP number tended to decrease caudally. A comparison of the DCS and NDCS groups revealed that the number of TCPs in the DCS group was significantly less than that in the NDCS group for C3, C4, and C5 (P < 0.05). The incidence of TCP at C6 in the DCS group was less than that in the NDCS group, but this difference was not significant (*P* > 0.05). In each group, the number of TCPs for male was less than that for female (P < 0.05).

### PTA

**Fig. 7:**
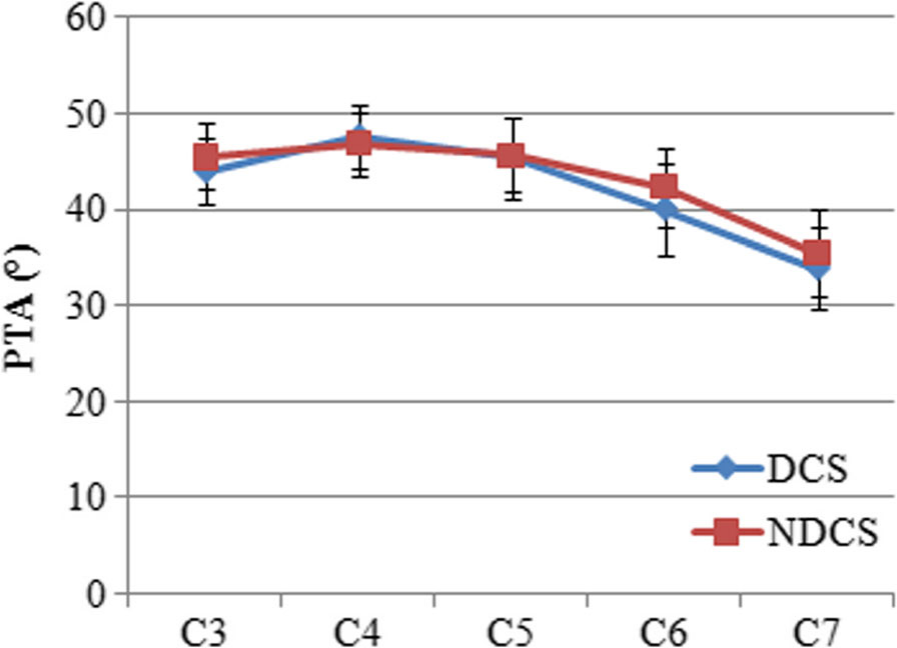
PTA in the DCS and NDCS groups. PTA indicated pedicle transverse angle

The mean PTA was 43.9 ± 3.5° for C3 and 33.8 ± 4.2° for C7 in the DCS group and 45.5 ± 3.4° for C3 and 35.9 ± 4.6° for C7 in the NDCS group (Table 2). The mean PTA tended to decrease from C4 to C7 in the both groups. The smallest mean PTA was found at C7, while the largest was at C4 in the both groups (Fig. 7). A comparison of the DCS and NDCS groups revealed that there was no significant difference at any level (*P* > 0.05). The PTA between male and female was no significant difference in each group.

### RSA

**Fig. 8:**
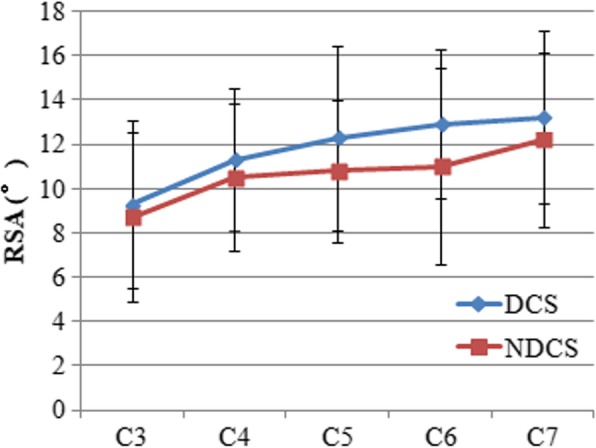
RSA in the DCS and NDCS groups. RSA indicated range of safe angle

The mean RSA was 9.3 ± 3.3° for C3 and 13.2 ± 3.9° for C7 in the DCS group, and 8.7 ± 3.2° for C3 and 12.2 ± 3.9° for C7 in the NDCS group (Table 2). As shown in Fig. 8, the mean RSA tended to increase from C3 to C7 in both groups. The smallest mean RSA was found at C3, while the largest was at C7 in both groups (Fig. 8). A comparison of the DCS and NDCS groups revealed the RSA in the DCS group was significantly greater than that in the NDCS group from C4 to C7 (*P* < 0.05). The RSA at C3 in the DCS group was greater than that in the NDCS group, but this difference was not significant (P > 0.05). In each group, the RSA for male was greater than that for female (P < 0.05).

## Discussion

Pedicle screw fixation provides effective stabilization with simultaneous decompression to the cervical spine, and is typically used to treat those patients with local kyphosis, segmental instability, or anterior conditions which had previously been operated on [5, 6, 19]. As DCS has been reported to occur at a high rate in patients with cervical spondylotic myelopathy or cervical spinal cord injuries [1, 2, 20, 21], the application of cervical pedicle screws may be of value in these DCS patients. The risk of neurovascular injury as a result of poor screw positioning, however, limits the application of CPS insertion [8, 9]. DCS is often caused by dysplasia of the rear attachment structures of the cervical spine in children [1, 22]. Most studies have found that DCS patients often exhibit short pedicle, flat lamina, and the overlap of the lamina and the rear edge of the lateral mass line in lateral radiographs [23, 24]. Some studies have further produced morphological comparisons of the cervical posterior structures between DCS and NDCS patients [16, 25, 26]. Wang Z et al. [25] found through CT-based measurements that the majority of evaluated parameters differed between DCS and NDCS patients in northeastern China. However, the safety of subaxial CPS insertion in DCS patients has not previously been clarified. Namely, if the short pedicle with narrow canal is relatively small, posterior pedicle screws insertion maybe more dangerous for these patients. If not, cervical pedicle screws maybe more safe for a short and wide pedicle.

We therefore performed morphological measurements on CT reconstruction images, comparing these measurements between DCS and NDCS patients grouped according to the Pavlov ratio, which is commonly used in clinical settings [18]. These measurements not only provide objective information regarding CPS insertion in DCS patients, but also provide insight into the question of whether CPS insertion in DCS patients is more difficult. Morphological features relevant to CPS insertion include linear parameters, such as pedicle width, pedicle height, and pedicle length as well as angular parameters, such as the pedicle transverse angle and the pedicle sagittal angle. Previous studies have shown that pedicle width was the primary determinant of the feasibility and safety of CPS insertion, while the pedicle transverse angle was more important than sagittal angle with respect to the potential for neurovascular complications [11, 15, 27]. In the present study, we therefore measured the pedicle width and transverse angle carefully. We additionally assessed the incidence of small pedicles with a POW < 4 mm, and we calculated the range of safe angle. As previous comparisons of these parameters between male and female patients have yielded consistent results [11, 15], gender differences were not focused in this study.

The mean overall cervical pedicle width values and trends in our study were similar to those in previous reports [11–16]. Onibokun et al. [11] found in a multiplanar CT anatomical assessment of 122 subjects that the overall pedicle outer width ranged from 4.7 to 6.5 mm, with a trend towards increasing caudally. Miyazaki et al. [16] found in a CT myelography analysis of 52 subjects comparing DCS and NDCS patients that the POW ranged from 4.8 to 5.9 mm in DCS patients and from 4.7 to 6.4 mm in NDCS patients. The mean POW in the DCS group was significantly less than that in the NDCS group at C6 and C7, with no significant differences at C3, C4, or C5. Unlike this latter study, we observed that the POW in DCS patients was wider than that in NDCS patients at every level. This may be due to the different classification methods used for establishing study groups. This previous studies classified study groups according to the spinal canal longitudinal diameter (SCLD), whereas we relied upon the Pavlov ratio. Moreover, racial variations may have contributed to these different study outcomes. Indeed, our results indicated that subaxial CPS insertion in DCS patients may be easier than that in NDCS patients.

The smallest CPS diameter available is 3.5 mm in clinical practice. As such, pedicles with a diameter of 4.0 mm were assumed to be the smallest into which a screw could feasibly be inserted, as documented in previous studies [15, 28]. In our study we identified instances of TCP in both patient groups from C3 to C6 (8.3 to 11.6% in the DCS group; 15.0 to 35.0% in the NDCS group), with no instances at C7. A comparative analysis revealed there were fewer TCPs in DCS patients from C3 to C5. Thus, preoperative CT scanning and trajectory planning is essential for safe and effective CPS insertion. Our study also indicated that greater consideration should be taken for pedicle insertion in NDCS patients owing to the greater number of cervical pedicles in these individuals.

Proper angulation is an important factor for CPS insertion [27, 29]. Injury to the medial breach may result in cerebrospinal fluid leakage or spinal cord injury, whereas injury to the lateral breach may cause vertebral artery injury. In our study, observed pedicle transverse angles closely paralleled those reported in previous studies [16, 30–32]. Tan et al. [32] reported an average PTA range of 30° to 46° from C3 to C7 in a study of Chinese Singaporeans, with the smallest average PTA at C7. Miyazaki et al. [16] found the transverse angles from C3 to C7 ranged from 30.6° to 41.9° in DCS patients, and from 30.8° to 42.4° in NDCS patients, with no significant difference between these groups. Our study supported the finding of these previous studies, revealing a similar PTA range of 35° to 47° in the DCS and NDCS groups. In addition, we further measured the maximum transverse angle, the minimum transverse angle, and the range of safe angle in the two groups included in our study. Our results revealed that the RSA in the DCS patients was greater than that in NDCS patients at all levels except for C3. However, this range of safe angle at every level in the two groups was very small (about 10°), even neglecting the diameter of CPS. That no significant differences were detectable at C3 may be due to the small POW at this level, resulting in limited change between the maximum and minimum transverse angles. In addition, we observed large individual variations in this study as evidenced by the relatively wide PTA range and the large standard deviation for each level. Given this wide degree of variability, a preoperative CT evaluation of the PTA is vital in all patients to ensure safe CPS insertion.

## Conclusions

Our study compared the morphological features of subaxial cervical pedicles between Chinese DCS and NDCS patients. We found that DCS patients had, on average, a greater pedicle outer width, a lower incidence of tiny pedicles (POW < 4 mm), and a larger range of safe transverse angle. Thus, transpediclar screw insertion in DCS patients may be safer and more feasible than it is for NDCS patients. However, as this study was retrospective in nature and limited to a single institution, future multicenter prospective studies which also control for other variables such as variations between ethnic groups are warranted. Moreover, subaxial cervical pedicles are relatively small, especially from C3 to C5, and pedicle insertion is not possible in pedicles smaller than 4 mm. As such, preoperative CT evaluation with trajectory planning is recommended for all DCS patients undergoing this procedure.

## Abbreviations

CD: Canal diameter
CPS: Cervical pedicle screw
CSIWFD: Cervical spinal cord injury without fracture and dislocation
CSM: Cervical spondylotic myelopathy
CSR: Cervical spondylotic radiculopathy
CT: Computed tomography
DCS: Developmental canal stenosis
Max TA: Maximum transverse angle
Min TA: Minimum transverse angle
NDCS: Non-developmental canal stenosis
PACS: Picture archiving and communication system
POW: Pedicle outer width
PTA: Pedicle transverse angle
RSA: Range of safe angle
SCLD: Spinal canal longitudinal diameter
SPSS: Statistical package for social sciences
TCP: Tiny cervical pedicle
VD: Vertebral diameters
